# Evaluation of physical activity programmes for the elderly - exploring the lessons from other sectors and examining the general characteristics of the programmes

**DOI:** 10.1186/1756-0500-4-368

**Published:** 2011-09-26

**Authors:** Ana I Marques, Pedro Soares, Luísa Soares-Miranda, Carla Moreira, António Oliveira-Tavares, Paula Clara-Santos, Susana Vale, Rute Santos, Joana Carvalho

**Affiliations:** 1 Research Centre in Physical Activity, Health and Leisure, Faculty of Sport, University of Porto, Porto, Portugal; 2 Department of Physical Education, José Estêvão High School, Aveiro, Portugal; 3 School of Health Technology of Porto, Polytechnic Institute of Porto, Porto, Portugal; 4 Research Centre in Sports, Health Sciences and Human Development, Higher Institute of Maia, Maia, Portugal

**Keywords:** physical activity, elderly people, quality, assessment, EFQM

## Abstract

**Background:**

In Portugal, there are several physical activity (PA) programmes for elderly people developed by the local government. The importance of these programmes has been increasing since the evidence has shown that this type of health promotion interventions may reduce the deleterious effects of the ageing process. However, no study has already identified the general characteristics of these programmes nor if they use any scheme to assess the quality of the service provided. A widely-used scheme is the EFQM Excellence Model, which will be in the core of our present work. Thus, the main aims of this preliminary study were 1) to identify the general characteristics of the PA programmes developed by the Portuguese Local Public Administration 2) to determine the extent of implementation of quality initiatives in these programmes.

**Methods:**

Data were collected by an on-line questionnaire sent to all Continental Municipalities (n = 278). Categorical data were expressed as absolute counts and percentages. Continuous data were expressed as the mean and SD. An open-ended question was analysed using qualitative content analysis with QSR NVivo software. Associations between categorical variables were tested by the use of contingency tables and the calculation of chi-square tests. Significance level was set at p ≤ 0.05.

**Results:**

Results showed: i) a total of 125 PA programmes were identified in the 18 districts of the Portugal mainland; ii) the main goal of the majority (95.2%) was the participants' health promotion; iii) different characteristics of the programmes were found according to different regions of the country; iv) certain characteristics of the programmes were associated to the existence of other features; v) only one PA programme developed quality initiatives.

**Conclusions:**

In conclusion, although there are many PA programmes for elderly people spread throughout the country, aiming at improving the health of participants, the overwhelming majority does not adopt quality control initiatives. Considering that the quality of a service increases customer satisfaction, the continuous quality improvement of the PA programmes for elderly people should therefore be implemented since they can be useful and critical for elderly satisfaction and adherence.

## Background

Biopsychosocial changes arising from the ageing process can negatively affect the quality of life of the elderly by limiting their ability to carry out everyday activities and exposing them to a greater vulnerability to health problems [1]. Evidence provided by several studies highlights that physical activity (PA) can play a major role on global health promotion [2,3], in large part by epidemiological evidence of the positive effect of an active lifestyle and involvement of individuals in PA programmes [4,5]. Indeed, these programmes are particularly important to prevent and minimize the deleterious effects of the ageing process [6,7] and to improve quality of life [1,6-8]. Nevertheless, a substantial proportion of European elderly adults -- with particular relevance to the Portuguese population -- have lower PA levels than those recommended for good health [9,10]. Therefore, increasing adherence to PA among elderly people is actually an important public health challenge. Several authors suggest that higher attendance in PA programmes is influenced by degrees of enjoyment and satisfaction [11-14]. Therefore, continuous quality improvement of the PA programmes for elderly people can be crucial for elderly satisfaction and adherence, since one of the most important factors for customer satisfaction is providing a quality service [15-17].

The *National Center for Chronic Disease Prevention's Division of Nutrition and Physical Activity *described a set of recommendations and strategies to improve programmes, developing new approaches and highlighting the need for effective programme evaluation [18,19]. Furthermore, programme evaluation is a useful tool for continuous quality improvement [20] and the WHO guidelines for the evaluation of health promotion emphasize the need to evaluate and propose the allocation of adequate resources for this action [21].

In Portugal, Public Administration is the sector that offers the largest supply of goods and services, and as such, should be the sector that must devote most attention to Quality and to the definition of quality standards. In this way, a quality management model is essential in order to improve the public service delivery to citizens and better allocate scarce public resources.

With the objective of helping organizations to improve their performance, the European Foundation for Quality Management (EFQM) introduced in 1991 the Excellence Model, which is currently used by thousands of organizations throughout Europe, such as companies, health institutions, schools, public safety services and governmental institutions, among others. The model also provides organizations with a common management vocabulary and tools, thus facilitating the sharing of best practices between organizations of different sectors [22].

**Figure 1:**
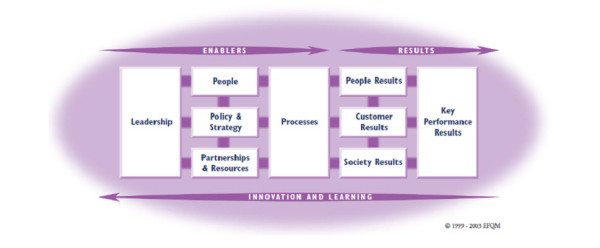
**EFQM Excellence Model (EFQM, 2003a)**.

The EFQM Excellence Model (Figure 1) is a non-prescriptive framework, based on nine criteria divided into thirty-two sub-criteria. Of these nine criteria, five are 'Enablers' - what an organization does to achieve excellence - and four are 'Results' - what an organization achieves, that is, the results achieved on the path to Excellence. 'Results' are caused by 'Enablers' and the feedback from 'Results' help to improve 'Enablers'. The arrows presented in the model show the dynamic nature of the model; the issues related to 'Innovation and Learning', while horizontal vectors essential for the model's architecture, emerge as cross-sectional elements in all the criteria. They show innovation and learning to improve enablers that in turn lead to improved results.

The model recognizes that there are many approaches to achieving sustainable Excellence in all aspects of performance, based on the premise that: "Excellent results with respect to Performance, Customers, People and Society are achieved through Leadership driving Policy and Strategy that is delivered through People, Partnerships and Resources, and Processes" [17]. Definitions of the Model criteria are given below, in Table 1.

**Table 1:** Definitions of the Model criteria (adapted from EFQM, 2003a).

MODEL CRITERIA	DEFINITION
**Leadership**	Excellent Leaders develop and facilitate the achievement of the mission and vision. They develop organisational values and systems required for sustainable success and implement these via their actions and behaviours. During periods of change they retain a constancy of purpose. Where required, such leaders are able to change the direction of the organisation and inspire others to follow.
**Policy & Strategy**	Excellent Organisations implement their mission and vision by developing a stakeholder focused strategy that takes account of the market and sector in which it operates. Policies, plans, objectives, and processes are developed and deployed to deliver the strategy.
**People**	Excellent organisations manage, develop and release the full potential of their people at an individual, team-based and organisational level. They promote fairness and equality and involve and empower their people. They care for, communicate, reward and recognise, in a way that motivates staff and builds commitment to using their skills and knowledge for the benefit of the organisation.
**Partnerships & Resources**	Excellent organisations plan and manage external partnerships, suppliers and internal resources in order to support policy and strategy and the effective operation of processes. During planning and whilst managing partnerships and resources they balance the current and future needs of the organisation, the community and the environment.
**Processes**	Excellent organisations design, manage and improve processes in order to fully satisfy, and generate increasing value for, customers and other stakeholders.
**Customer Results**	Excellent organisations comprehensively measure and achieve outstanding results with respect to their customers.
**People Results**	Excellent organisations comprehensively measure and achieve outstanding results with respect to their people.
**Society Results**	Excellent organisations comprehensively measure and achieve outstanding results with respect to society.
**Key PerformanceResults**	Excellent organisations comprehensively measure and achieve outstanding results with respect to the key elements of their policy and strategy.

It is around these nine criteria and the thirty-two sub-criteria that an organization's progress towards excellence is assessed. Self-assessment will shed light on the areas requiring improvement and how to conduct improvement actions, acting on the process.

The implementation of the EFQM Excellence Model within the Public Administration has been principally publicised within the healthcare sector, with its inherent benefits largely discussed by Jackson [23]. Several authors [24-30] have also discussed the implementation of the excellence model within health and social care environments.

Furthermore, many approaches have been made in education institutions, especially in the higher education system. Models based on quality awards such as the EFQM Excellence Model or models created for self-assessment in academia, have become an important instrument to implement self-assessment methodology for quality improvement in higher education institutions [31,32].

In the last years, particular attention has been devoted to this framework by the local governance sector. In order to achieve the quality plan goals, Bologna Municipality top managers chose to employ the EFQM Excellence Model in 1997 [33] and this action was followed by many other cities of Europe [34,35].

Additionally, there has been a growing concern about quality and quality management within the public leisure services, which has resulted in the introduction of quality programmes and its associated techniques, such as EFQM Excellence Model, to facilitate leisure management [36-38]. Robinson highlighted the significant role played by quality management as an appropriate strategy for the management of public leisure facilities in bringing about a customer-focused approach to service delivery and the evidence of its assignment in improving service quality [36]. The research carried out by the same author [37] indicated that nearly one third of public leisure facilities use the EFQM Excellence Model for the reason that its use led to improvements in service, primarily through clearer procedures and continuous improvement.

Taking into account that, in Portugal, there are several PA programmes for elderly people developed by the local government, involving many employees and activities that reach thousands of participants and also expend considerable public fees, it seems appropriate a quality assessment of these PA programmes. However, to our knowledge, there is no specific tool to assess the quality of the service provided. Thus, the main aims of this preliminary study were 1) to identify the general characteristics of the PA programmes developed by the Portuguese Local Public Administration and 2) to determine the extent of implementation of quality initiatives in these programmes.

## Methods

An *on-line questionnaire *was sent out to all Portuguese Continental Municipalities (n = 278) in May, 2008. This questionnaire has provided the following information: geographic localization, number of programmes to enhance quality of life for elderly people (name and objectives), age of the PA programme [39], characteristics of age groups and participants' average age [40,41], number of activities offered in the PA programme [42,43], frequency of the programme (days/week) [1,39], quality initiatives [20,44-47], name of the organization that delivers the programme, and identification details of the PA programme's coordinator (Additional file 1). Question format ranged from closed questions with multiple choices and dichotomous type to open-ended question. Categorical data were expressed as absolute counts and percentages. Continuous data were expressed as the mean and SD.

An open-ended question which addressed the objectives of the programme was analysed using qualitative content analysis with QSR NVivo software. Contingency tables and chi-square tests were used to analyse associations between categorical variables, performed with the Statistical Package SPSS, version 17.0. Significance level was set at p ≤ 0.05.

## Results and Discussion

### Number of PA programmes and geographic localization

**Figure 2:**
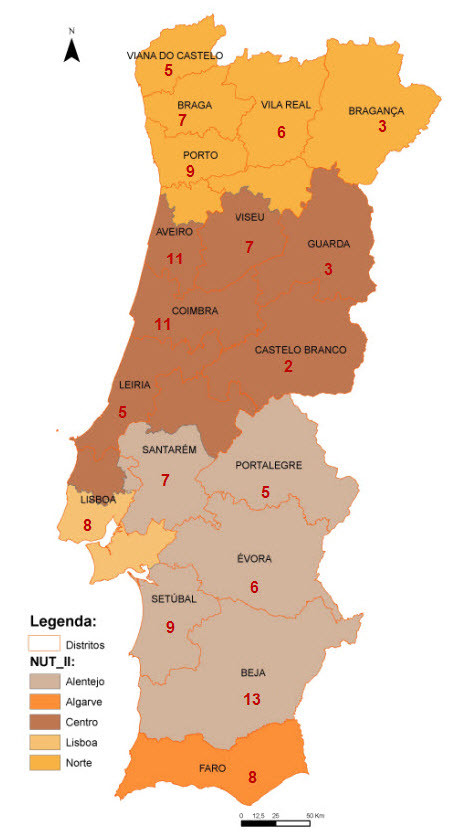
**Distribution of PA programmes by district; representation of NUT_II**.

**Figure 3:**
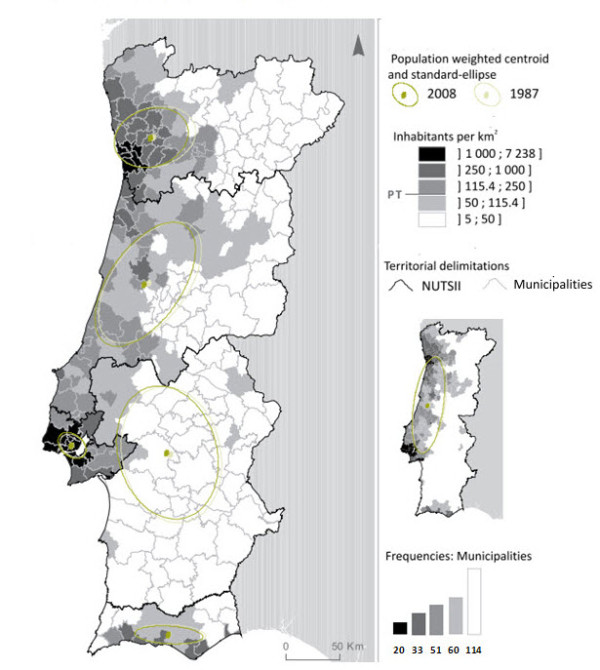
**Population density by municipality and by NUT_II (INE 2009)**.

**Figure 4:**
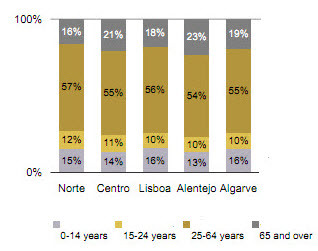
**Distribution of resident population according to age groups, by NUT_II (INE 2009)**.

Of the 278 municipalities, 97 questionnaires were totally answered. Since some municipalities provided more than a single programme, 174 programmes intended to enhance the quality of life for elderly people were identified. Of these, 125 were PA programmes. Figure 2 represents the geographical distribution of the 125 PA programmes in the 18 districts of the Portugal mainland and it also represents the 5 regions (NUT_II). The largest percentage of programmes was located in the littoral districts of the Continent (58.9%) where there is the greatest number of residents and more percentage of individuals aged 65 or more, i.e., 69.4% [48], as revealed in Figure 3 and Figure 4.

### Objectives of the PA programme

The major objective focused was "to promote health" (95.2%) remotely followed by "to improve physical function" (28.8%), "to create socialization opportunities" (25.6%) and "to prevent disease" (18.4%), as reflected in Table 2. Chi-square analysis showed a higher than expected number of programmes that aim "to create socialization opportunities" in the Alentejo region, possibly due to the fact that this places are separated by vast plains of uninhabited territory, with a poor transport network and a lack of opportunities for socialization [49], which are generally located in more urbanized localities. On the contrary, the programmes belonging to the North are those that give less value to this objective (p = .017). Instead, it is in the North of the country that the programmes pay more attention to the objective "to promote physical activity" (p = .04). When analyzing the national territory according to the coastland areas and inland areas, we found that the programmes from the coastland give more importance to the objective "to improve self-esteem/self-confidence" (p = .023). In line with this diversity of objectives found in the PA programmes of the present study, scientific evidence supports that regular PA has several physical, psychological and social beneficial effects on a variety of health outcomes [1,6,50-54].

**Table 2:** Objectives of the PA programmes for elderly people

	%	n
To promote health	95.2	119
To prevent disease	18.4	23
To improve physical function	28.8	36
To create socialization opportunities	25.6	32
To promote social recognition	9.6	12
To improve self-esteem/self-confidence	11.2	24
To promote leisure occupation	15.2	19
To promote physical activity	16.8	21

### Age of the PA programme

**Figure 5:**
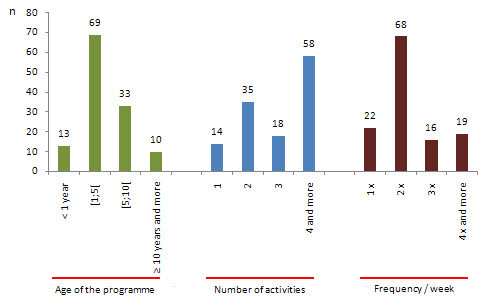
**Characteristics of PA programmes' age, number of activities and frequency/week**.

The results (Figure 5) indicate that the most common age of the PA programme was "one year of age and less than five", representing 55.2% and "five years of age and less than ten", representing 26.4%. Programmes with 10 or more years (8%) are located mainly in the Lisbon region, possibly due to the fact that there is a greater concentration of population aged 65 years or more (p ≤ .000) [48]. This may have led Lisbon region's politicians to be sooner concerned than their peers regarding the design of programmes that meet the elderly people' needs. This has been made easier possibly because of the presence of town halls' organizational structures necessary for the development of programmes, such as sports divisions, and qualified people with a degree in physical education or sport [55]. In addition, programmes that are located on the coastland (also with the highest concentration of elderly population) are those that are established for longer (p ≤ .000). The fact that many programmes have emerged in recent years may suggest that local government has made an effort to create initiatives aimed at increasing PA in elderly adults, integrating issues of ageing into social and local health policies. It will also be noted that since the late nineties, the Municipal enterprises of sport have expanded with increasing impact [56], providing favourable conditions for the development of these programmes. Moreover, the global tendency toward the decentralization of policies, which also includes those concerning the promotion of PA and the implementation of effective health-promotion strategies with regard to the distribution and administration of resources, highlights the key role that must be played by local authorities [57].

### Characteristics of age groups and participant's average age

Table 3 provides the characteristics of age groups, i.e. minimum and maximum age required to enrol in the PA programme, and the average age of participants.

**Table 3:** Characteristics of age groups and participant's average age

	minimum age intended				maximum age intended					
	55	60	65	70	75	80	85	90	95	n.l.
**%**	68	16.8	14.4	0.8	4.8	11.2	2.4	16	1.6	64
**n**	85	21	18	1	6	14	3	20	2	80

**mean ± SD **participants' age					72.23 ± 1.54					

n.l.: not limited

While the maximum age intended is, in most cases, "not limited" (64%) and the minimum age is 55 years (85%), the average age of participants is 72.23 ± 1.54 years. The majority of programmes have a minimum age of 55 years as a pre-requisite for admission (68%), followed by those who require 60 years as the minimum age (16.8%). Some authors [58-60] advocate that the benefits of sufficient aerobic exercise, even if started as late as age 60 years, is associated with a 1-to-2 year increase in life expectancy as well as improved functional independence and quality of life benefits. According to a six-year study carried out by researchers at the US National Institute on Aging, elderly people who are physically active are much more likely to live longer than elderly people who are not physically active [61]. However, there are still 19 programmes (15.2%) that have higher minimum ages (65 and 70 years old). The available data from the Contemporary Portugal Database [40] indicates that the oldest age group (75+) increased at the fastest pace (from 1991 to 2001, their number increased 32.8% from 527948 to 701366). Actually, people's life expectancy in developed countries has increased greatly over the last 25 years, leading to an increase in the retirement age [41]. Shephard argues that in early old age (65-75 years), there may be a modest increase of PA, in an attempt to fill free time resulting from retirement [62]. In this way, the minimum age required to enrol in some PA programmes, although high, respond to demographic and social trends.

### Number of activities offered in the PA programme

Figure 5 gives an overview of the number of activities offered in PA programmes. The majority number of activities was "4 or more", representing 46.4%, followed by the PA programmes with "two activities", which reached 28%. Programmes with 10 or more years are those that offer more activities, while programmes with 1-5 years offer two activities (p = .003). These results suggest that older programmes are more aware of recommendations concerning this issue. Roberts and Brodie suggest that such programmes should offer a wide range of activities, while allowing individuals to focus on those gradually that they identify as more likely to engage in regularly [42]. Among other considerations, the AHA scientific statement [43] also stated that these programmes should fulfil the demands of different needs between women and men, embrace occupational and leisure activities and simple tasks of daily living, incorporate the importance of socialization and include a diversity of exercise activities to enhance PA participation of the elderly. Simultaneously, our data suggest that more recent programmes seem to be more cautious regarding the inclusion of different activities, preferring initially to get a deeper understanding of customer needs.

### Frequency of the programme (days/week)

The usual frequency with which individuals participate in the overall programme are two times per week (Figure 5), representing 54.4%. Moreover, 28% of the programmes allow seniors to sessions for three or more times per week, offering them organized opportunities to be physically active. Programmes of 6 to 10 years of age are those that can be attended a greater number of days per week (p = .034). Consequently, the international recommendations [6] to increase the level of PA among older people in order to reach at least 30 minutes or more of moderate-intensity PA on most -- preferably all -- days of the week are more easily achieved. The Lisbon region presents a larger than expected number of programmes with a weekly frequency of three times, while the Centro region presents a greater number of programmes that could be frequented only once per week (p = .006). When compared the number of activities offered by the programme with the weekly frequency, it was observed that the greater the number of activities, the greater the number of days per week that an individual can participate in the programme (p = .003).

### Quality initiatives

Just one PA programme for elderly people (0.8%) has quality initiatives, in this particular case, a quality management system certification. Beyond certification, the certifying institution provides customized solutions to increase the quality and efficiency of the programme. The use of quality schemes in public leisure services in Portugal [38] is widely divergent from use in other countries [37,63-65], where their governments act directly in this matter. In this respect, several studies [37,44-46] found that the quality initiatives may improve process and outcomes. The *Healthy Ageing - A Challenge for Europe Report *[47] suggests a systematic application of quality management/assurance methods to increase project's quality; these indicate that Quality is an important issue for PA programmes for older people. Simultaneously, the Benchmark 3 from Physical Activity and Health Branch at the CDC [20] advocate a complete programme evaluation in order to improve their continuous quality improvement. This reinforcement is given by the CDC with the following statement: *'the evaluation is the systematic examination and assessment of features of an initiative and its effects, in order to produce information that can be used by those who have an interest in its improvement or effectiveness' *(CDC 2002 [19], p.5). So, in opposition to what was found in the present study, it seems that PA programmes for elderly people must be assessed to make informed decisions when planning new initiatives or examining existing services, in order to improve them. It also reveals commitment to delivering the highest quality service viable with available resources.

### Organization that delivers the programme

The main organization that owns the programme was the "municipal government" (85.6%) distantly followed by "other" (7.2%) and "municipal enterprises of sport" (4.8%), as reflected on Table 4. The municipal governments are responsible for developing programmes in the Lisbon region, whereas in the Alentejo region, the Local City Centre or other local organizations develop them (p = .005). These results suggest that in regions where there is greater dispersion of the population, such as Alentejo [48], governments and other organizations closer to the population are responsible for developing these programmes, revealing a greater involvement of different partners. The development and sustainment of the community partnerships is the first public health benchmarks for PA Programmes established by the Physical Activity and Health Branch at the CDC [20].

**Table 4:** Organization name

	%	n
Municipal Government	85.6	107
Municipal enterprises of sport	4.8	6
Local City Centre	2.4	3
Other	7.2	9

## Conclusion

In conclusion, data showed that the majority of the 125 PA programmes identified in the present study set the goal of promoting the health of participants, which reflect the current recommendations. Furthermore, the majority of programmes have a minimum age of 55 years as a pre-requisite for admission. However, there are still some programmes that have higher minimum ages. It was also observed that the greater the number of activities, the greater the number of days per week that an individual can participate in the programme, with most of the programmes offering two activities and having a frequency of two times per week. The "municipal government" was the main organization responsible for developing the PA programmes.

Moreover, in spite of an eminent preoccupation with health, quality of life and autonomy of older subjects inherent to the PA programmes studied, there is no effective use of quality initiatives, seen as an important process to improve programmes. Indeed, our results showed that only one PA programme develops quality initiatives. In summary, the results of the present study highlight the need of continuous quality improvement of the Portuguese PA programmes for elderly people, since it can be critical for elderly satisfaction and adherence.

In closing, although these findings provide some clues, future research may be needed to characterise the quality management models of the PA programmes developed by the Portuguese Local Administration, using the EFQM' criteria or other tool considered applicable.

## Strengths and Limitations

To our knowledge, this was the first study exploring the general characteristics of the Portuguese PA programmes for elderly people, as well as identifying which organizations were developing quality initiatives. The relevance of this investigation is that it offers a direction for further research into quality management in an area that has not previously been extensively examined.

However, a major limitation is worthy of comment. Taking into account that the invitations to participate in the study were done online, so the answer to the questionnaire was voluntary, care should be taken in extrapolating our findings, since our sample is, probably, not representative of all PA programmes developed in Portugal.

## Competing interests

The authors declare that they have no competing interests.

## Authors' contributions

AIM participated in the acquisition and analysis of data and participated in drafting and editing the manuscript. PS supervised the drafting and editing of manuscript. LSM and CM managed the data collection and analysis. AOT provided technical support on the data collection and analysis. PCS and SV helped design the questionnaire and managing the online process. RC and JC participated in the coordination of the study and supervised the drafting and editing of manuscript.

All authors read and approved the final manuscript.

## Supplementary Material

Additional file 1 **On-line questionnaire**. Explanation of the structure and content of the on-line questionnaire Click here for file
