# Supplementary material for: Evaluation of physical activity programmes for the elderly - exploring the lessons from other sectors and examining the general characteristics of the programmes
Source: BMC Res Notes. 2011 Sep 26;4:368. doi: 10.1186/1756-0500-4-368 (PMC3197507; doi:10.1186/1756-0500-4-368)
Supplement: Additional file 1 — On-line questionnaire. Explanation of the structure and content of the on-line questionnaire [file 1756-0500-4-368-S1.PDF]

ONLINE QUESTIONNAIRE

WARNING: Please do not reply on this page. Access http://www.ensaios.org/inquerito1/ and enter the code of your municipality (you can copy and paste). Thank you!

1 - Geographic location (automatic)

2 – Is there any programme aiming to enhance quality of life for elderly people in your municipality?

○ NO, ○ YES

3 – Please, indicate the number of existing programmes:

(Tables with questions 3.1 and 3.2 will be generated according to the number given)

3.1 - Programme 1

Title:

Main Purpose:

3.2 – Is the programme related to physical activity / exercise?

○ NO, ○ YES

(*Based on the response to this question, the questionnaire continued to question 4 or skipped to the final online page -- using conditional branching)*

4 – How long has the programme existed?

○ Less than 1 year, ○ 1 year - less than 5 years, ○ 5 years - less than 10 years, ○ 10 years or more

5 – For what age group is this programme intended? (Tick the boxes that most closely represent the intended lower and upper age limits)

Minimum: ○ 55 years, ○ 60 years, ○ 65 years, ○ 70 years, ○ 75 years, ○ 80 years, ○ 90 years

Maximum: ○ 70 years, ○ 75 years ○ 80 years, ○ 85 years, ○ 90 years, ○ 95 years, ○ no limit

6 – What is the average age of participants attending the programme (approximately)?

7 - How many activities are included in the programme?

○ 1, ○ 2, ○ 3, ○ 4 +

8 – How many times per week is it possible for an individual to participate in the programme?

○ 1, ○ 2, ○ 3, ○ 4 +

9 – Is the programme involved (or was) in quality initiatives (ex.: programme certification)?

○ NO, ○ YES

10 – Which organization delivers the programme?

○ Municipal Government, ○ Municipal enterprises of sport, ○ Other

11 – Please, specify the name of the organization which delivers the programme and indicate the name, role, and contact information of the director/coordinator of the programme for possible future contacts.
